# Molecular Drivers of Platelet Activation: Unraveling Novel Targets for Anti-Thrombotic and Anti-Thrombo-Inflammatory Therapy

**DOI:** 10.3390/ijms21217906

**Published:** 2020-10-24

**Authors:** Madhumita Chatterjee, Agnes Ehrenberg, Laura Mara Toska, Lisa Maria Metz, Meike Klier, Irena Krueger, Friedrich Reusswig, Margitta Elvers

**Affiliations:** 1 Department of Cardiology and Angiology, Universitätsklinikum Tübingen, Medizinische Klinik III, 72076 Tübingen, Germany; madhumita.chatterjee@med.uni-tuebingen.de; 2 Department of Vascular and Endovascular Surgery, Experimental Vascular Medicine, Heinrich-Heine University Medical Center, 40225 Düsseldorf, Germany; agnes.ehrenberg@hhu.de (A.E.); laura.toska@hhu.de (L.M.T.); lisa.metz@hhu.de (L.M.M.); mekli100@uni-duesseldorf.de (M.K.); irena.krueger@uni-duesseldorf.de (I.K.); Friedrich.reusswig@uni-duesseldorf.de (F.R.)

**Keywords:** platelets, integrins, glycoprotein Ib, glycoprotein VI, reelin, phospholipase D, CXCR, pannexin 1, NMDAR

## Abstract

Cardiovascular diseases (CVDs) are the leading cause of death globally—partly a consequence of increased population size and ageing—and are major contributors to reduced quality of life. Platelets play a major role in hemostasis and thrombosis. While platelet activation and aggregation are essential for hemostasis at sites of vascular injury, uncontrolled platelet activation leads to pathological thrombus formation and provokes thrombosis leading to myocardial infarction or stroke. Platelet activation and thrombus formation is a multistage process with different signaling pathways involved to trigger platelet shape change, integrin activation, stable platelet adhesion, aggregation, and degranulation. Apart from thrombotic events, thrombo-inflammation contributes to organ damage and dysfunction in CVDs and is mediated by platelets and inflammatory cells. Therefore, in the past, many efforts have been made to investigate specific signaling pathways in platelets to identify innovative and promising approaches for novel antithrombotic and anti-thrombo-inflammatory strategies that do not interfere with hemostasis. In this review, we focus on some of the most recent data reported on different platelet receptors, including GPIb-vWF interactions, GPVI activation, platelet chemokine receptors, regulation of integrin signaling, and channel homeostasis of NMDAR and PANX1.

## 1. Introduction

Cardiovascular diseases (CVDs) include ischemic heart disease, stroke, heart failure, peripheral arterial disease, and a number of other cardiac and vascular conditions. They are the leading cause of death globally. Every year, more people die from cardiovascular disease than any other cause, with 31% of deaths following cardiovascular diseases worldwide [1,2,3].

Platelets are small anucleate cells of the hematopoietic system and are formed by megakaryocytes (MKs) in the bone marrow. At sites of vascular injury, platelets adhere to the injured vessel wall, forming a hemostatic plug to avoid excessive blood loss, and therefore play a fundamental role in hemostasis. In contrast, platelets trigger thrombotic events because uncontrolled platelet activation can induce acute vessel occlusion, leading to myocardial infarction or stroke at areas of atherosclerotic plaque rupture [4,5]. Ischemic CVDs such as myocardial infarction and stroke as well as infectious diseases are characterized by thrombotic and inflammatory events that contribute to both cell death and organ failure. This thrombo-inflammation is mediated by platelets and immune cells such as T cells, macrophages and neutrophils and triggers organ dysfunction [6,7,8].

Injury of the endothelium leads to exposure of extracellular matrix proteins that serve as substrates for adhesion and activation of circulating platelets. Platelet activation and thrombus formation include different signaling pathways leading to platelet shape change, integrin activation and degranulation. Platelet recruitment to the vessel wall and initial adhesion is dependent on the interaction between glycoprotein (GP) Ib-IX-V and von Willebrand factor (vWF), that binds to subendothelial collagen [9]. In the second step, platelets become activated characterized by shape change, activation of integrins and secretion [10]. In the third step, platelet adhesion to the vessel wall is stabilized followed by platelet aggregation and thrombus formation. These latter processes are mainly mediated by integrins [11], but the mechanisms involved are not fully understood. Thus, many efforts have been made to identify new molecular targets for anti-thrombotic and anti-thrombo-inflammatory therapy in the last years.

## 2. GPIb-vWF-Axis

During vascular injury, subendothelial matrix proteins such as collagen, fibronectin, thrombospondin and endothelial released vWF are exposed to the blood stream. Platelet surface exposed receptors are able to bind to these sub-endothelial matrix components to avoid massive blood loss. A first, transient binding of platelets to the injured vessel wall is achieved by binding of GPIb, exclusively expressed on the platelet surface, to collagen-adherent vWF, thus tethering platelets to the vessel wall. However, binding of GPIb to vWF is characterized by a rapid on-off rate that facilitates binding of the collagen receptor GPVI to fibrillar collagen in the injured vessel wall to initiate platelet activation, firm adhesion and thrombus formation [12]. Under high shear conditions, GPIb-vWF interaction is essential for the recruitment of platelets to the injured vessel wall [5] and initiates intracellular signaling cascades involving tyrosine phosphorylation of SYK, the Src family kinases Lyn and Fyn, and phospholipase (PL) Cγ2 to induce α_IIb_β_3_ integrin activation and firm adhesion [13,14,15]. Thus it is not surprising that inhibition of the GPIb-vWF axis could be a promising target for therapy in ischemic stroke to improve cerebral reperfusion rates and reduce cerebral injury after ischemia and reperfusion as demonstrated by Denorme et al. [16].

### Reelin and PLD1 Are New Regulators of GPIb Signaling

**Figure 1:**
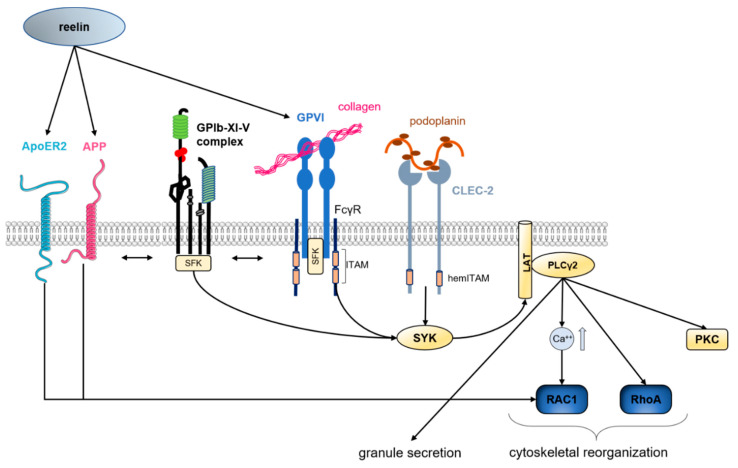
Impact of reelin in platelet activation and ITAM signaling. Extracellular reelin binds to ApoER2 and APP receptors at the platelet surface, resulting in platelet activation through RAC-1 activation. Additionally, reelin binding indirectly modulates GPIb signaling through APP and GPVI. Ligand binding to GPVI leads to dimerization of GPVI monomers, inducing auto-transphosphorylation of the GPVI-associated FcγR-chain ITAM motif through activation of Src-family kinases (SFKs). Besides, podoplanin binding to the CLEC-2 receptor causes activation of the CLEC-2-integrated hemITAM motif. Phosphorylated ITAM motifs lead to phosphorylation of SYK, which activates LAT kinases, being in direct proximity to PLCγ2. Activated PLCγ2 causes granule release, activation of protein kinase C (PKC), and activation of RAC1 and RhoA through calcium influx leading to cytoskeletal reorganization. This figure was created using images from Servier Medical Art Commons Attribution 3.0 Unported License. (http://smart.servier.com). Servier Medical Art by Servier is licensed under a Creative Commons Attribution 3.0 Unported License”.

In recent years, different modulators of GPIb activation and signaling have been identified. The glycoprotein reelin has been shown to be important for GPIb-dependent thrombus formation [17]. Reelin was first identified in the central nervous system where it plays a prominent role in brain development by controlling neuronal positioning and migration to mediate the formation of laminated brain structures. It is produced by Cajal-Retzius neurons and interacts with the lipoprotein receptors apolipoprotein E receptor 2 (ApoER2), mediating the formation of filopodia and lamellipodia, and with the very-low-density lipoprotein receptor (VLDLR) [18,19]. Reelin is found in blood plasma and is expressed in endothelia cells, in the liver [20,21] and in megakaryocytes and platelets, where it co-localizes to F-actin and regulates platelet spreading on fibrinogen [17,22]. Recent study identified reelin as a new promising therapeutic target to prevent occlusive thrombus formation. Reelin-deficient mice are protected against arterial thrombosis and focal cerebral ischemia but show normal hemostasis. Reelin deficient platelets display reduced adhesion to recombinant vWF under high shear conditions but exhibit no defects in vWF binding under static conditions, when binding of vWF to GPIb is induced by the snake venom botrocetin. After GPIb engagement, integrin activation and the phosphorylation of Erk and Akt are reduced in reelin deficient platelets. Reelin-induced GPIb activation is mediated by the interaction of GPIb and the major platelet reelin receptor amyloid precursor protein (APP) suggesting that reelin-induced effects on GPIb signaling are mediated by APP–GPIb interactions (Figure 1) [17].

The family of PLD, including the most prominent isoforms PLD1 and PLD2 [23], catalyzes the degradation of phosphatidylcholine into choline and phosphatidic acid (PA) [24], representing a very important messenger in many cellular processes [25]. An important role for PLD1 in GPIb-dependent thrombus formation under flow conditions has been shown using PLD1-deficient mice. Loss of PLD1 in platelets impairs integrin α_IIb_β_3_ activation and GPIb-dependent aggregate formation, thereby protecting against thrombosis and ischemic brain infarction without altering hemostasis in PLD1-deficient mice [26]. In contrast, the loss of PLD2 does not affect platelet activation [27,28]. Recent studies provided evidence for yet another important role of PLD1 in platelet-mediated inflammation [29]. According to the prominent role of GPIb in the adhesion of platelets to endothelial cells (ECs) via binding to selectins under high shear rates [30], PLD modulates the up-regulation of endothelial adhesion molecules and regulates the adhesion of platelets to ECs. In addition, PLD1 contributes to platelet-leukocyte recruitment under inflammatory conditions. Thus, PLD1 plays a role in thrombo-inflammatory processes known to aggravate organ damage following ischemia/reperfusion injury after ischemic stroke and myocardial infarction [26,31]. Indeed, in an experimental model of myocardial infarction, loss of PLD1 leads to defective cell adhesion and migration of inflammatory cells into the infarct border zone, and to altered scar formation resulting in enhanced infarct size and declined myocardial function [31]. Recently, a role for PLD2 in platelet-mediated inflammation was identified. PLD2-deficient mice exhibit enhanced IL-6 plasma levels concomitant with enhanced migration of inflammatory cells into the infarct border zone 24 h after acute myocardial infarction. This was due to enhanced integrin α_IIb_β_3_ activation of PLD2-deficient platelets under inflammatory conditions, resulting in enhanced IL-6 release of ECs to accelerate inflammation after myocardial infarction, suggesting that PLD2 is an effector of thrombo-inflammation [28].

These results emphasize the important role of the vWF-GPIb axis not only in hemostasis and thrombosis but also in thrombo-inflammatory processes following ischemia and reperfusion injury after stroke and myocardial infarction contributing to organ damage. Therefore, the identification of novel modulators of GPIb signaling such as reelin and PLD1 is of utmost interest and might represent promising therapeutic targets to prevent thrombosis and thrombo-inflammation without inducing bleeding complications.

## 3. ITAM-Signaling Pathways

Loss of the immunoreceptor tyrosine-based activation motif (ITAM)-coupled receptors glycoprotein (GP) VI or C-type lectin-like receptor (CLEC)-2 is linked to only a mild bleeding diathesis in patients as well as in mice [32,33,34]. Both receptors share the conserved immunoreceptor tyrosine-based activation motif (ITAM) sequence containing four amino acids. A tyrosine separated from an (iso)-leucine by two other amino acids, called the YXXL motif. The GPVI/Fcγ chain consists of two cytosolic YXXL motifs. In contrast, CLEC-2 consists of only a singular cytosolic YXXL motif, thus named hemITAM [35,36,37].

### 3.1. GPVI Signaling

GPVI is the major receptor for collagen and is expressed on megakaryocytes and platelets. Surface plasmon resonance studies revealed that fibrous collagen binds to dimeric but not to monomeric GPVI via the glycine-proline-hydroxyproline (GPO) sequence [38]. GPVI activation leads to Src family kinase-mediated phosphorylation of the two ITAMs and induces the binding and phosphorylation of SYK. Phosphorylated SYK in turn induces the phosphorylation of the adaptor protein LAT and the recruitment of different pathway effectors resulting in the activation of PLCγ2. The activation of PLCγ2 by phosphorylation triggers the mobilization of intracellular Ca^2+^ stores as well as the activation of PKC. These events lead to the release of intracellular α-granules and dense granules and inside-out activation of platelet integrins, resulting in platelet aggregation [37].

In recent years, several GPVI ligands beside collagen have been identified including fibrin, diesel exhaust particles (DEP) and large polysaccharides such as fucoidan and dextran sulfate [39,40,41]. Interestingly, GPVI was shown to bind to polymerized fibrin to amplify thrombin generation and to recruit additional platelets to the thrombus. Thus, GPVI is a receptor for fibrin, supports phosphatidylserine (PS) exposure and promotes thrombus growth and stability [42].

Since GPVI is a key receptor involved in the pro-thrombotic stage of acute coronary syndrome (ACS) it is presumed to be a useful biomarker for the early detection of atherosclerotic diseases and may contribute to risk stratification and prediction of clinical outcome in patients with ACS [43,44,45,46,47]. Furthermore, soluble GPVI may serve as a target for molecular imaging to identify vulnerable plaques [48]. In addition to the property of GPVI as a diagnostic tool, therapeutic implications have been developed such as the blockage of collagen binding sides of GPVI by a soluble recombinant GPVI-Fc protein. This protein binds to collagen upon vessel injury and avoids binding of platelets via membrane bound GPVI to the injured vessel. Furthermore, anti-GPVI antibodies are useful to block platelet aggregation in static blood [49,50,51,52,53,54]. Treatment of mice with GPVI-Fc or the bifunctional protein consisting of an stromal cell-derived factor 1 (SDF-1) domain and a GPVI domain (SDF-1-GPVI) preserved cardiac function in a mouse model of myocardial infarction [55,56]. Thus, therapeutic anti-GPVI strategies could serve as a promising strategy for anti-thrombotic and anti-atherosclerotic therapy.

#### Reelin Amplifies GPVI Signaling in Platelets

Recently, reelin was identified as a novel regulator of GPVI signaling beside its regulatory role in GPIb signaling and shear-dependent thrombus formation.

Reelin is released after platelet activation with collagen and binds to GPVI with sub-nanomolar affinity in a concentration-dependent manner to amplify GPVI-dependent signaling in platelets [57]. In detail, reelin binding to GPVI induces tyrosine phosphorylation of GPVI target proteins to support platelet binding to collagen and GPVI-dependent RAC1 activation, PLCγ2 phosphorylation and platelet aggregation (Figure 1). Antibody-mediated deletion of GPVI from the platelet surface in reelin-deficient mice completely abolishes thrombus formation in vivo. Thrombus formation is only partly reduced in either GPVI-depleted or reelin-deficient mice. Thus, interfering with reelin-GPVI interaction might be a novel strategy to avoid arterial thrombosis [57].

### 3.2. CLEC-2 Signaling

The ability of CLEC-2 monomers to form homodimers is essential for signal transduction. To date podoplanin expressed on lymphatic endothelium and on tumor cells but not on vascular endothelial cells, is identified as the endogenous ligand found for CLEC-2. During podoplanin-mediated platelet aggregation, glycosylated podoplanin interacts with CLEC-2, leading to receptor clustering and phosphorylation of the hemITAM motifs. Moreover, although it is well known that GPVI phosphorylation of the ITAM motif is induced by the Src family kinases (SFK) Fyn and Lyn, it is still controversial how CLEC-2 is initially phosphorylated. Until now, several models of CLEC-2 hemITAM phosphorylation are published. Severin et al., demonstrated that—in contrast to GPVI activation—the tyrosine kinase SYK mediates CLEC-2 phosphorylation independently of the SFKs Fyn, Lyn or Src [58]. In contrast, others postulated that a phosphoinositide 3-kinase (PI3K)/tyrosine-protein kinase Tec (Tec)-axis regulates SYK activation downstream of CLEC-2 [59] and that CLEC-2 phosphorylation is initiated by SFKs. However, once recruited, phosphorylated SYK induces the engagement and phosphorylation of the adaptor protein LAT similar to GPVI. From this point, GPVI and CLEC-2 share the same signaling cascade leading to platelet aggregation and secretion. To date, podoplanin is the only known natural ligand for CLEC-2. However, some studies report increased tail bleeding times and reduced thrombus formation in CLEC-2-deficient mice, suggesting the presence of an additional ligand in the vasculature [60,61].

In the past, different reports provided evidence for GPVI or CLEC-2 to be a major player in platelet-mediated processes of inflammation. Transfer of GPVI or CLEC-2-deficient platelets to thrombocytopenic mice revealed an important role for ITAM-coupled receptors in inflammatory hemostasis of the skin and the lung [36]. In a mouse model of stroke, GPVI was identified as a key player in the processes of neuronal damage following cerebral reperfusion injury [49,62]. Similarly, antibody-mediated deletion of GPVI or treatment of mice with recombinant SDF-1-GPVI reduced inflammation and infarct size demonstrating a pivotal role of GPVI in ischemia reperfusion injury following ligation of the left anterior descending artery (LAD) in a mouse model of myocardial infarction [56,63]. More recently, it was shown that the inhibition of GPVI reduced the adhesion of amyloid beta (Aβ)-activated platelets to injured carotid arteries in mice suggesting a role of GPVI in Aβ-mediated inflammation in Alzheimer’s disease [64].

Only a few studies investigated the role of CLEC-2 in inflammatory processes suggesting that CLEC-2 is a major player in thrombo-inflammatory diseases. Inflammatory events in the vessel wall characterize deep vein thrombosis (DVT) where reduced thrombosis was observed in CLEC-2 deficient mice or after treatment of mice with an anti-podoplanin antibody [65]. So far, there is only experimental evidence for a role of CLEC-2 in thrombo-inflammation in mice. However, Nicolson et al. detected up-regulated podoplanin in the venous valves near to a femoral vein thrombus in a patient with DVT. Interestingly, no alterations in podoplanin expression have been detected in the unaffected valves of the same vein or the equivalent valves in the contralateral leg [66].

## 4. G-Protein Coupled Receptor Activation—CXCRs as Emerging Modulators

G-Protein-coupled-receptors (GPCRs) are seven-pass transmembrane receptors that transduce intracellular signals through physical interaction with heterotrimeric G-proteins [67,68,69], e.g., G_s_, G_i_, G_q_, and G_12/13_, located at the cytoplasmic face of the plasma membrane [70,71]. Platelets express a number of GPCRs that are directly engaged by activating stimuli, protease-activated-receptors (PAR) for thrombin, purinergic receptors-P2Y12, P2Y1 for adenosine diphosphate (ADP), thromboxane receptor (TP) for thromboxane A_2_ (TxA_2_) or by physiological inhibitors (prostaglandin I_2_ receptor (IP) for prostaglandin I_2_ (PGI_2_) [72]) released from the vasculature [67,73]. PAR1 and PAR4 transduce signals through G*α*_q_ and G*α*_13_; P2Y_1_ is coupled to G*α*_q_; P2Y_12_ is coupled to G*α*_i_ [74,75,76,77], while the TxA_2_ interacting TP*α* receptor is coupled to G*α*_q_ and G*α*_13_ in platelets [78]. Therefore, these GPCR are top candidates for therapeutic intervention [71,73,79]. On the other end of the spectrum arachidonic acid (AA)-derived PGI_2_, also called prostacyclin, acts as a physiological anti-platelet agent by engaging G*α*_s_-coupled IP receptor. Prostaglandin D_2_ (PGD_2_) activates G*α*_s_-coupled prostaglandin D_2_ receptor 1 (DP_1_)_,_ which triggers the platelet inhibitory adenylyl cyclase-protein kinase A cascade [69,72,80]. Prostaglandin E2 (PGE_2_) exhibits a biphasic, dose-dependent effect through EP_1_, EP_2_, EP_3_ and EP_4_ receptors either activating or inactivating platelet response [67,69,80]. Recent research in search of novel anti-platelet targets to overcome the challenge of bleeding complications associated with some of the GPCR antagonist in clinical practice (vorapaxar) have revealed some unconventional targets that may fine-tune platelet reactivity.

### CXCR Chemokine Receptors on Platelets Fine-Tune Thrombosis

**Figure 2:**
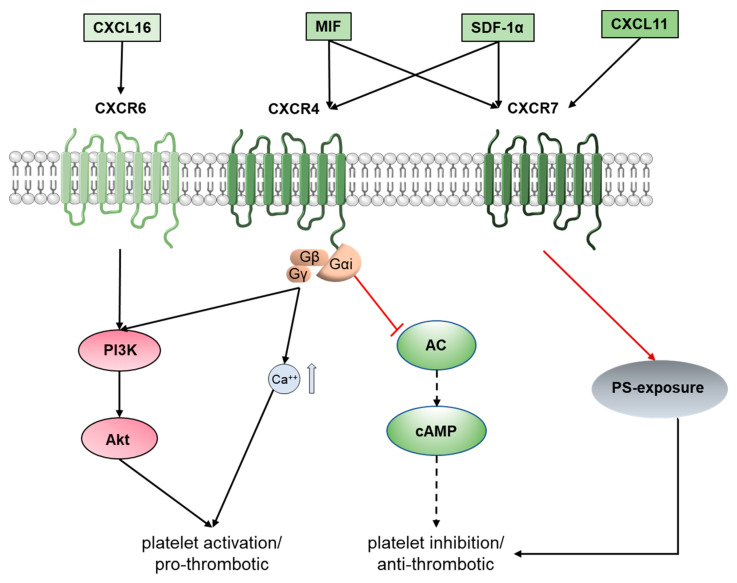
Regulation of platelet activation by CXCR chemokine receptors. Different chemokine receptors like CXCR6, CXCR4 and CXCR7 induce GPCR signaling in platelets. The chemokines CXCL16, and CXCL12/SDF-1α activate the phosphatidylinositol 3-kinases (PI3K) pathway leading to the activation of protein kinase B (Akt) and calcium mobilization resulting in platelet activation. Simultaneously, AC is inhibited through Gαi downstream of CXCR4 ligation by CXCL12/SDF-1α. MIF binding to CXCR7 substantiates platelet survival through PI3K-Akt pathway and downregulates phosphatidyl serine (PS) exposure. This figure was created using images from Servier Medical Art Commons Attribution 3.0 Unported License. (http://smart.servier.com). Servier Medical Art by Servier is licensed under a Creative Commons Attribution 3.0 Unported License”.

Platelet chemokine receptors (e.g., C-C chemokine receptor type 1 (CCR1), CCR3, CCR4, CXC receptor 2 (CXCR2), CXCR4, CXCR6 and CXCR7) are a category of GPCRs that aggravate or reduce thrombotic response [81,82,83,84,85,86,87,88] (Figure 2). Platelets derived chemokines, e.g., C-X-C motif chemokine ligand 12 (CXCL12), CXCL16, and the cytokine macrophage migration inhibitory factor (MIF), released upon activation, engage with their cognate receptors and modulate thrombotic attributes, while in a paracrine manner elicit thrombo-inflammatory response from CXCR4, CXCR6, CXCR7 expressing immune and vascular cells [88,89,90,91,92,93]. Soluble CXCL16, acting through CXCR6 on platelets, triggers the PI3K-Akt activating signaling cascade to substantiate aggregation, degranulation, integrin α_IIb_β_3_ activation and platelet shape change [88] (Figure 2). Serum levels of CXCL16 are significantly elevated in ACS patients as compared to those with stable angina pectoris (SAP) [94]. They correlate with inflammatory and metabolic risk factors [95], are shown to be prognostically unfavorable [96,97] and independently associated with cardiovascular death and morbidity in ACS patients [98] enrolled in the PLATO (platelet inhibition and patient outcome) trial. Surface expression of scavenger receptor (SR-PSOX/CXCL16) for the atherogenic mediator oxidized low density lipoprotein (oxLDL) on platelets is significantly enhanced in ACS patients and correlates with the circulatory inflammatory marker C-reactive protein (CRP) and creatinine kinase (CK) [99]. CXCL16 is abundantly deposited on inflammatory atherosclerotic plaques and carotid endarterectomy specimens and promotes CXCR6-dependent platelet adhesion to the inflamed endothelial cells and vWF [100], suggesting its potential to propagate thrombo-inflammation.

The Gi-coupled canonical CXCR4 and atypical/non-canonical CXCR7 are platelet receptors for the pro-inflammatory chemokine SDF-1α/CXCL12 and the chemokine-like cytokine MIF, whereas interferon inducible T cell alpha chemoattractant (ITAC, also named CXCL11) exclusively ligates CXCR7 [101,102] (Figure 2). Moreover, the presence of ligands in the immediate microenvironment influences a dynamic alteration in CXCR4/CXCR7 surface expression in platelets [101,102]. Platelet CXCR4/CXCR7 surface expression is significantly elevated in coronary artery disease (CAD) patients and influences prognosis [87] following myocardial infarction (MI) [86,87,103]. Mechanistic insights reveal the functional dichotomy of CXCR4 and CXCR7 in substantiating thrombotic response through CXCR4, whereas a pro-survival and anti-thrombotic impact was observed through CXCR7 [101,103]. MIF does not alter degranulation, ADP- or TxA2-analog (U46619)-induced aggregation or spreading on fibrinogen [91,92,101], either alone or synergistically in combination with other agonists. However, the pro-survival effect of MIF-CXCR7, significantly attenuates the exposure of thrombogenic phospholipid phosphatidylserine and counteracts thrombus build up [101] (Figure 2). On the contrary, CXCL12/SDF-1 acting through Gi-coupled CXCR4 clearly exerts a pro-thrombotic impact (Figure 2). Platelets exhibit flow directed migration towards CXCL12/SDF-1 [104] and also transmigrate through endothelium following a CXCL12 gradient, attributed to CXCR4 [105]. Once in a CXCL12/SDF-1 enriched microenvironment, e.g., atherosclerotic plaques, the CXCL12/CXCR4-Gi axis in platelets triggers intracellular calcium mobilization, PI3K activation, while dampening platelet inhibitory cAMP levels, leading to augmented aggregation [7,13,14,15,16,17,106,107,108,109] and thrombotic potential (Figure 2). SDF-1α-CXCR4 localized to lipid rafts in the platelet membrane trigger platelet reactivity [110], while decreased surface expression of CXCR4 in patients with inherited thrombocytopenia corroborates with impaired CXCL12/SDF1α-triggered platelet aggregation [111]. CXCL12 induces the primary phase of aggregation at lower concentrations, but dose dependently instigates a biphasic aggregation profile involving PI3K and prostanoids [107]. CXCL12 also synergistically substantiates aggregation induced by sub-threshold concentration of ADP and thrombin [108] and relatively weaker agonist serotonin (5HT), an effect substantiated by granular release (ADP, adenosine triphosphate (ATP)) [109], P-selectin exposure [108], PLC activation, and release of TxA_2_ [109]. CXCL12 increases platelet adhesion to collagen type IV and fibrinogen, an effect counteracted by apyrase [108] and mediated through ATP release, TxA_2_ production, promoting aggregation and thrombus formation [109,112], in contrast to the anti-thrombotic influence of MIF [101] (Figure 2).

Plasma levels of CXCL12/SDF1α [113] and MIF [114] are elevated in ACS patients and associated with progressive disease severity, which may influence platelet responsiveness differentially through CXCR4/CXCR7. Platelet CXCR4 surface expression correlates inversely whereas CXCR7 correlates positively with inflammatory and atherogenic oxLDL in platelets from CAD patients. CXCR4 and CXCR7 also mediate the synergistic effects of pro-inflammatory CXCL12 on LDL and oxLDL induced changes to platelet morphodynamics and functional response [112], highlighting the potential of CXCL12/CXCR4/CXCR7 in executing thrombo-inflammatory platelet functions in a hyperlipidemic environment to promote atheroprogression. Differences in release kinetics and specific trigger for secretion may direct the course and pattern of functional impact exerted by different chemokines on platelet response as they engage CXCR6, CXCR4, or CXCR7. These GPCRs may provide promising therapeutic alternatives to check thrombotic functions without compromising the hemostatic and regenerative capacity of platelets.

## 5. Integrin Signaling in Platelets

### 5.1. Integrin Structure

Integrins are heterodimeric transmembrane proteins expressed on the cell surface of different cells. They serve as adhesion receptors that trigger intracellular signaling pathways by binding extracellular ligands [115]. Integrins are composed of two non-covalently bound subunits [115,116]. Overall, there are eight β- and 18 α-chains known. Through different combinations of these various subunits, 24 integrins have been identified in mammals that can bind a variety of adhesion ligands [117,118]. Due to their function as adhesion receptors, integrins mediate the adhesion to the extracellular matrix and between cells. Therefore, they are involved in processes like cell differentiation, proliferation, and migration in organisms, as well as in platelet adhesion and aggregation [115,117,119].

Five different integrins are described at the platelet surface. The classification of these integrins is based on the different β-subunits. There are three β_1_- and two β_3_-class integrin receptors exposed at the platelet surface. Members of the β_1_-class integrin receptors are the collagen receptor α_2_β_1_, the fibronectin receptor α_5_β_1_ and the laminin receptor α_6_β_1_. The fibrinogen receptor α_IIb_β_3_ and the vitronectin receptor α_v_β_3_ correspond to the β_3_-class integrin receptors (Table 1) [118].

The most redundant and prominent integrin receptor on platelets is the fibrinogen receptor integrin α_IIb_β_3_ (also known as GPIIb/IIIa). About 80,000 of these glycoprotein complexes can be found in their inactive state on the surface of resting platelets [120]. Furthermore, the membrane of α-granules expresses α_IIb_β_3_ integrins. Activation-induced secretion of α-granules leads to externalization of α_IIb_β_3_ integrins at the platelet surface and serves as a marker of platelet activation [121]. Rare mutations in the integrin subunits have been shown to modify the interaction between the subunits of the integrin. This connection is formed by a salt bridge that links the intracytoplasmic part of αIIb to the β3 unit of the α_IIb_β_3_ integrin. Mutations in the salt bridge cause abnormal proplatelet formation with abnormal large α-granules and reduced but not absent platelet aggregation [122].

### 5.2. Bidirectional Signaling of Integrin α_IIb_β_3_: Role of Paxillin, Reelin and PLD1

**Figure 3:**
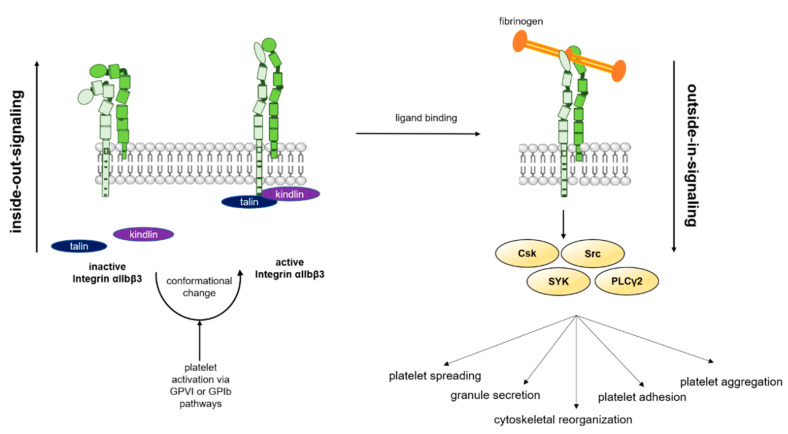
Inside-out- and outside-in-signaling of integrin α_IIb_β_3_. In resting platelets, integrin α_IIb_β_3_ is in an inactive conformation that prevents ligand binding. However, an initial activation of platelets (e.g., via GPIb or GPVI signaling) leads to binding of cytosolic kindlin and talin. This binding induces a conformational change of integrin α_IIb_β_3_ that allows the binding of fibrinogen and other ligands. The process which induces the conformational change of integrin α_IIb_β_3_ is called inside-out signaling. Binding of a ligand like fibrinogen to the extracellular domain of integrin α_IIb_β_3_ triggers a signaling cascade through various kinases. This amplifies different platelet processes such as spreading, granule secretion and aggregation. The process of extracellular ligand-induced activation of integrin α_IIb_β_3_ is called outside-in signaling. This figure was created using images from Servier Medical Art Commons Attribution 3.0 Unported License. (http://smart.servier.com). Servier Medical Art by Servier is licensed under a Creative Commons Attribution 3.0 Unported License”.

In the plasma membrane of platelets, integrin α_IIb_β_3_ serves as bidirectional receptor for inside-out and outside-in signaling [115]. The inside-out signaling is induced by binding of different soluble or immobilized agonists to platelets (e.g., thrombin, ADP, TxA_2_, epinephrine, vWF, collagen, etc.) via GPCRs or GPs such as GPIb or GPVI at the platelet membrane. Platelet activation triggers intracellular signaling cascades that lead to a conformational change of integrin α_IIb_β_3_ [117]. The binding of the intracellular protein talin to the cytoplasmic part of integrin α_IIb_β_3_ results in an unclasping of the cytoplasmic and transmembrane domain of α_IIb_ and β_3_. This unclasping finally triggers a conformational change of the extracellular domain. The talin-triggered conformational change of integrin α_IIb_β_3_ is supported by the interaction of kindlin and the C-terminal region of the β3 cytoplasmic domain [123,124,125]. Investigating the mechanistic details, Gao et al. recently showed that kindlin directly interacts with paxillin. The disruption of this binding through mutations in the binding site significantly impaired the activation of integrin α_IIb_β_3_. This suggests that the interaction between kindlin and paxillin supports talin-mediated integrin α_IIb_β_3_ activation that could be a target for anti-thrombotic therapies [126]. The conformational change of the α_IIb_β_3_ integrin activates the receptor from its inactive into its active state [115]. This activation drives binding of ligands with higher affinity (Figure 3) [117]. Fibrinogen as major ligand binds to α_IIb_β_3_ via its HHLGGAKQAGV sequence that is located in the C-terminus of the γ-chain of fibrinogen and the RGD sequence in the α-chain of integrin α_IIb_β_3_. Since the RGD sequence can be found in other proteins, such as vitronectin, fibronectin, and von Willebrand factor, it is not surprising that these proteins are ligands that can be bound to α_IIb_β_3_ integrin as well [125].

The binding of ligands to the extracellular domain of integrin α_IIb_β_3_ induces integrin clustering and promotes integrin outside-in signalling [115]. Integrin outside-in signaling triggers a set of intracellular processes like platelet spreading, cytoskeleton reorganization, clot retraction, granule secretion as well as platelet adhesion and aggregation that finally leads to thrombus formation and stabilization [127]. During integrin α_IIb_β_3_ (α_5_β_1_ and α_2_β_1_)-mediated platelet adhesion, the protein disulfide isomerase (PDI) inherits an important role as enzymatic mediator for disulfide exchange [128]. Lahav et al. could show that specific blockers of PDI inhibit the aggregation of platelets [129]. In mice, quercetin-3-rutinoside block platelet accumulation as well as fibrin generation at the vascular injury site [130]. In patients who are at risk of thrombosis, quercetin flavonoids inhibit PDI activity in plasma and reduce platelet-dependent thrombin generation [131].

Regulation of integrin α_IIb_β_3_ outside-in signaling involves different interacting proteins, which associate with the cytoplasmic tails of α_IIb_β_3_. The tyrosine kinase c-Src comes into proximity to the active cytoplasmic tail of the β_3_ integrin subunit to induce full catalytic activation by trans-autophosphorylation to initiate outside-in signaling [127,132]. This is followed by coordinated interactions of Csk, Src, and SYK kinases with integrin α_IIb_β_3_ to induce integrin signaling finally resulting in cytoskeletal reorganization [133]. Furthermore, a Src-kinase-dependent activation of PLCγ2 and phosphorylation of MLC mediates clot retraction downstream of integrin α_IIb_β_3_ [134]. Enhanced integrin α_IIb_β_3_ outside-in signaling was observed in mice with a gain-of-function mutation in PLCγ2 [135]. Platelets from these mice show accelerated spreading on different matrices and elevated clot retraction suggesting a major role for PLCγ2 in integrin outside-in signaling.

Based on the finding that ibrutinib can inhibit platelet integrin α_IIb_β_3_ outside-in signaling, Dobie and colleagues investigated the effects of this inhibitor on platelet activation and glycoprotein expression in more detail by [136]. Ibrutinib induced the shedding of integrin α_IIb_β_3_ from the platelet surface in a time- and dose-dependent manner as detected by reduced integrin α_IIb_β_3_ surface expression of platelets isolated from patients with chronic lymphocytic leukemia, who were treated with ibrutinib. The underlying molecular mechanisms including the responsible sheddases are still unknown [137]. PLD1 modulates platelet α_IIb_β_3_ integrin activation; therefore reduced shear-dependent thrombus formation in PLD1-deficient mice was observed [26]. More recently, Klier et al., could show reduced platelet adhesion to endothelial cells caused by defective integrin activation confirming a role for PLD1 not only in hemostasis and thrombosis but also in platelet-mediated inflammation [29]. Analyzing the impact of reelin signaling in platelets and its role for integrin activation and thrombus formation, Gowert et al. showed that reelin is a mediator of GPIb-dependent integrin activation as well [17]. In contrast to cytoplasmic PLD1 that affects integrin signaling by the activation of Src [29], reelin binds to its main receptor APP at the platelet surface leading to the co-localization of APP and GPIb to induce integrin activation. However, the molecular mechanisms behind the impact of reelin on integrin activation are not entirely clear to date.

## 6. Polymorphonuclear Leukocyte Released Platelet Agonists

Activated platelets release pro-inflammatory chemokines and cytokines and show an increased expression of P-selectin and CD40-ligand on their surface. These platelets are able to interact with leukocytes and endothelial cells leading to the recruitment of neutrophils into the inflamed tissue [138]. Interaction of polymorphonuclear leukocytes (PMNLs) with platelets is important for host defense and is associated with an increased risk of thrombosis induced by inflammatory pathways. The ability of PMNLs to release antimicrobial peptides, so called human neutrophil peptides (HNPs) or α-defensins, is an important mechanism of the immune response. Once released, HNPs are able to destroy microorganism membranes to exhibit their antimicrobial activity [139]. Besides their role in host defense, it was shown that HNPs act as platelet agonists, leading to platelet degranulation and even shedding of microparticles. Horn et al. showed that HNPs are able to induce platelet apoptosis by the formation of amyloid-like structures. Moreover, HNPs induce the formation of polymeric fibrinogen and thrombospondin-1 structures where platelets adhere and form aggregates. Furthermore, microorganisms are captured by these amyloid-like structures thus linking thrombosis and infection. Blocking of integrin α_IIb_β_3_ (GPIIb/IIIa) strongly inhibits HNP-induced activation of platelets [140].

Recently, it was shown that transgenic mice expressing human α-Def-1 (Def^++^) developed larger occlusive neutrophil-rich clots after ligation of the inferior vena cava (IVC), characterized by abnormal fibrin networks. Moreover, these mice were resistant to thrombo-prophylactic treatment with heparin. The inhibition of HNP synthesis or release was able to rescue this phenotype showing smaller thrombi and restoration of the responsiveness to heparin [141].

These observations explain an increased thrombotic risk at sites of inflammation and provide further insights into mechanisms linking inflammation to thrombosis. Glycosaminoglycans and serpins, which are potent inhibitors of platelet activation by HNPs, should be evaluated for therapeutic use in diseases characterized by activated hemostasis and inflammation [140]. Furthermore, drugs that inhibit neutrophil degranulation could be useful to treat or prevent thrombosis that develop during systemic inflammation with enhanced neutrophil activation [141].

## 7. Ion Channels: N-methyl-d-aspartate Glutamate Receptor (NMDAR) and Pannexin-1 (PANX1) as Modulators of Platelet Function

Ion homeostasis in each cell is a fundamental regulator of cellular functions and protection against apoptosis. In the last decades, the role of different ions was extensively investigated and Ca^2+^ was identified as a critical second wave mediator in different cell types [142]. Platelet activation in response to different agonists trigger different signaling pathways to mobilize cytosolic Ca^2+^ [143]. This process is essential for the reorganization of the cytoskeleton, the so called shape change [144]. Rapid increase in cytosolic Ca^2+^ is mediated by two main sources: (i) release of Ca^2+^ by an endomembrane system called dense tubular system (DTS) or (ii) by entry of Ca^2+^ through the plasma membrane [145]. Moreover, recent studies provide evidence that acidic organelles such as lysosomes are involved in the uptake and release of Ca^2+^, proposing a role for acidic organelles in platelet-mediated Ca^2+^ signaling [146]. Here, we focus (i) on the N-methyl-d-aspartate glutamate receptor (NMDAR) on the platelet membrane as well as (ii) on pannexin-1 (PANX1), an ion channel that mediates platelet activation and thrombus formation and how they potentially interact to mediate platelet responses.

### 7.1. N-methyl-d-aspartate Glutamate Receptor (NMDAR)

The N-methyl-d-aspartate glutamate receptor (NMDAR) belongs to the ionotropic glutamate receptor family and functionally consists of a heterotetramer or heteropentamer. Each receptor is composed of the essential NMDA receptor 1 (GluN1) subunit, a various number of NR2 subunits: GluN2A, GluN2B, GluN2C and GluN2D [147] and rarely GluN3 (A and B) subunits [148]. NMDAR plays a critical role in neuronal signaling and are activated by binding of its main agonists L-glutamate and glycine to the GluN1 and GluN2 subunit. Ligand-gated opening removes the Mg^2+^ plug which finally leads to Ca^2+^ exchange to maintain Ca^2+^ homeostasis [149].

**Figure 4:**
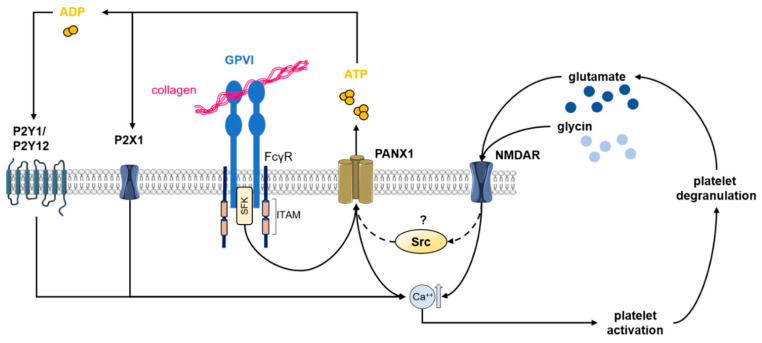
PANX1 and NMDAR activation upon platelet activation and signaling. Collagen binding induces activation of Src-family kinases (SFKs) which in turn activates PANX1 channels. Activation of PANX1 leads to an exchange of calcium and ATP, both modulating platelet activation through intra-and extracellular pathways. ATP directly binds to P2X1 or is degraded to ADP and binds to the purineric receptors P2Y1/P2Y12, all inducing platelet activation. Additionally, NMDAR might influence PANX1 activation through Src kinases, but this remains unclear in platelets to date. Besides, NMDAR activation induces calcium mobilization resulting in platelet activation. In a feedback loop, activated platelets release glutamate, an essential ligand for NMDAR activation. This figure was created using images from Servier Medical Art Commons Attribution 3.0 Unported License. (http://smart.servier.com). Servier Medical Art by Servier is licensed under a Creative Commons Attribution 3.0 Unported License”.

Platelets store about 400 µM glutamate in their dense granules which is released upon platelet activation and aggregation [150]. High extracellular glutamate concentrations lead to Na^+^ influx mediated by α-amino-3-hydroxy-5-methyl-4-isoxazolepropionic acid (AMPA) and kainite receptors contributing to membrane depolarization [150]. This process might enable L-glutamate binding to the GluN1 and GluN2 subunit to remove the Mg^2+^ plug leading to NMDAR activation and increased Ca^2+^ conduction in platelets (Figure 4).

Using immunocytochemistry, the group of Genever first described the expression of the initial GluN1 subunit on the platelet surface, but their function remains unclear [151]. In 2004, Kalev-Zylinska and colleagues identified the four subunits: GluN1, GluN2A, GluN2D and GluN3A that form the NMDARs on the platelet surface. Interestingly, the fully functional receptor complex is transported to the plasma membrane after platelet activation. This supports the hypothesis of a potential role of NMDARs in platelet mediated aggregation and thrombus formation [152]. However, human platelet aggregation was only slightly reduced in the presence of the NMDAR antagonist MK-801. A few years later, the same group demonstrated a role of NMDARs in promoting dense granule release, thrombus formation and stabilization, using an inhibitory antibody against the GluN1 subunit [153]. These data provide evidence that NMDARs might be involved in Ca^2+^ homeostasis upon platelet aggregation and thrombus formation. However, Ca^2+^ measurements with platelets from GluN1 knock-out mice have to be performed in the near future to clarify the role of NMDARs in intraplatelet calcium homeostasis and thrombotic responses.

### 7.2. Pannexin-1 (PANX1)

20 years ago a new subgroup of transmembrane proteins, called pannexins (PANX1, PANX2 and PANX3), were identified in many mammalian tissues [154]. PANX1 is ubiquitously expressed, whereas PANX2 is mostly found in the central nervous system (CNS) and PANX3 in bone and skin [155]. All three isoforms function as ion channels for small molecules with a high affinity for ATP. In 2014, Taylor and colleagues identified transcripts for PANX1, but not PANX2 and PANX3 in human platelets. Immunochemistry analysis revealed that PANX1 is predominantly located on the surface of platelets. Targeting PANX1 channels by probenecid (Prb) in vitro revealed that platelet activation upon stimulation with low concentrations of collagen induces opening of PANX1 channels and thereby amplifies Ca^2+^ influx and aggregation by activation of the P2X1 channel [156,157]. The activation of PANX1 channels via collagen is mediated by GPVI, leading to the phosphorylation of SFKs (Figure 4) [157]. Inhibition of SFKs reduces PANX1-dependent platelet aggregation as well as intracellular phosphorylation of PANX1 at Tyr308 [158]. Ex vivo thrombus formation on collagen under arterial shear rates is reduced when whole blood is treated with Prb [156]. More recently, the impact of platelet PANX1 in thrombosis and hemostasis has been confirmed using platelet-specific PANX1 knockout mice (Panx1(fl/fl)/PF4-cre+) that display extended bleeding times and defective arterial thrombosis in vivo [158].

In neuronal tissue, activated NMDARs cross-activate PANX1 through phosphorylation of SFKs [159]. Moreover, inhibiting Src kinase-mediated phosphorylation of PANX1 at Tyr308 blocks metabotropic NMDAR signaling in brain slices. Furthermore, disturbing the NMDAR-Src-PANX1 complex has been found to be neuroprotective after ischemia or stroke, suggesting that the NMDAR–PANX1 axis might be involved in thrombo-inflammation [160].

## 8. Conclusions

The high burden of CVDs and the impact of platelets in thrombotic and thrombo-inflammatory processes indicate a high medical need for effective and novel anti-thrombotic and anti-thrombo-inflammatory therapies that avoid thrombotic and inflammatory events while preserving hemostasis.

Platelet receptors have been exploited as therapeutic targets since decades. However, residual platelet reactivity and incidences of bleeding among susceptible individuals often lead to fatal consequences as evident from the outcome of several clinical trials conducted with FDA-approved antiplatelet agents, like P2Y12 antagonists [161] clopidogrel (in CAPRIE, CURE, CREDO, CLARITY, CHARISMA), prasugrel (in TRITON, TRILOGY), ticagrelor (in PLATO and PEGASUS), and competitive inhibitors of PAR-1 against thrombin, i.e., vorapaxar (TRACER [162] and TRA2P [163,164,165]). Currently, the choice of anti-platelet therapeutics in clinical practice is fairly limited to irreversible COX-1 inhibitor aspirin (ASA) for primary prevention of cardiovascular disease and dual anti-platelet therapy (ASA in combination with reversible (ticagrelor, cangrelor) and irreversible (clopidogrel, prasugrel) inhibitors of P2Y12) for secondary prevention of recurrent thrombotic events following acute coronary syndrome (ACS) or in patients with coronary stent implantation. Clinical trials, like PEGASUS-TIMI54 [166,167], DAPT [168,169,170], OPTIDUAL [171,172], COMPASS [173,174,175], and GEMINI-ACS1 [176], reflect their drawbacks in increasing non-fatal/fatal bleeding and falling short of expectations. These trials have validated the potential of single (aspirin), dual and triple anti-platelet therapy or anti-platelet therapy administered in combination with anti-coagulants (rivaroxaban) [176,177]. The therapeutic goal is to ascertain the cornerstone of antithrombotic treatment following ACS.

It is essential to prevent recurrent thrombo-ischemic events with efficient thromboprophylactic strategies and reassess the benefit-to-risk ratio [168,169,170,171,172,178,179], dosage regimens, and treatment duration. Novel anti-thrombotic mediators which can discriminate between the physiological processes of thrombosis and hemostasis may potentially achieve this fine balance between anti-thrombotic efficacy and bleeding risk [180]. Of these, specific inhibitors of PI3Kp110β [181] and PKC [182,183,184], besides tyrosine kinase inhibitors widely recommended against cancer, like SYK, BTK, those of the Src family [185,186], agents antagonizing integrin α_IIb_β_3_ by specifically targeting the active form of the receptor [187,188] or the outside-in signaling [189,190], a fusion protein combining the extracellular domain of CD39, and a single-chain antibody (Targ-CD39) that specifically detects activated integrin α_IIb_β_3_ [191] have emerged in recent years. Therapeutics utilizing CD39 [192,193,194], GPVI [195], and GPIb-IX-V [180] as targets/mediators have been of prime interest. These novel therapeutic targets have been effectively validated in animal models of thrombosis where they were administered before an experimental induction of thrombotic/thrombo-ischemic events, while PI3Kβ-inhibitor [196] and soluble GPVI (Revacept^®^) are in clinical trials. Some of these strategies like those against tyrosine kinases may exhibit off-target adverse effects on other cells or organs requiring restricted use in limited dose and over a limited time period. The potential anti-thrombotic mediators discussed in this review may exert significant influence on thrombotic and thrombo-inflammatory platelet function as these cells interact with the inflamed vascular bed or inflammatory cells, but essentially, these mediators are not prime drivers of platelet function. They may offer a fine-tuning adjustment in modulating important signaling events in platelets to retain physiological hemostasis but check pathological thrombosis. Additionally, potential anti-thrombo-inflammatory benefits of emerging anti-platelet approaches, like those targeting platelet secretion, e.g., inhibitors of platelet lysosome-derived protein disulfide isomerase (PDI) [197,198], recombinant ectonucleases that degrade the platelet agonist ADP [199], platelet-derived soluble P-selectin antagonist as validated in the SELECT-ACS trial [200] and those discussed in this review may be of vital significance in the prevention of thrombo-inflammatory complications associated with ischemic stroke [8], atherosclerosis [201], and other CVDs [202,203] in combating target organ damage following a thrombo-ischemic episode, recurrent thromboembolic events, and in-stent thrombosis. Anti-inflammatory effects of aspirin alone or in combination with P2Y12 antagonists, also rivaroxaban have been seen in clinical settings and in animal models [201]. Most of these beneficial effects stem from reduction in levels of inflammatory mediators like C-reactive protein, IL-6, TNF-α, MCP-1, IL-1β. The anti-inflammatory benefits of IL-1β targeting monoclonal antibody canakinumab, in the CANTOS-trial [204], in significantly reducing the rate of recurrent cardiovascular events affirm a promising future. Platelets are an active source of thrombo-inflammatory mediators [82,205]. The significant association between plasma IL-1β levels and circulatory platelet count, also platelet single-nucleotide polymorphisms (SNPs) assessed in GWAS, while inverse association between platelet count and plasma IL-1β antagonist α-1-anti-trypsin, signify the thrombo-inflammatory potential of platelets in CVD [206]. In this light novel therapeutics with additional impact on thrombo-inflammatory processes will certainly have an edge over conventional anti-platelet therapies in clinical practice. However, several important aspects need to be contemplated and verified for a translational implication. The differences in structure and functional response of animal (most often murine) and human platelets, the type and surface availability of receptors, and interspecies differences in the physiological processes of thrombosis-hemostasis are of particular significance. Nevertheless, constant preclinical and clinical research endeavors in delineating the molecular mechanisms dissecting hemostatic and thrombotic or thrombo-inflammatory platelet functions will present more options for therapeutic interventions and broaden the currently limited choice of anti-platelet strategies.

## Figures and Tables

**Table 1:** Platelet integrins with classification, glycoprotein (GP) nomenclature and corresponding ligands.

Classification	Integrin	GP Nomenclature	Ligands
β1-class	α_2_β_1_	GPIa/IIa	collagen
	α_5_β_1_	GPIc/IIa	fibronectin
	α_6_β_1_	GPIc‘/IIa	laminin
β3-class	α_IIb_β_3_	GPIIb/IIIa	fibrinogen, fibronectin, thrombospondin, vitronectin, von Willebrand factor
	α_v_β_3_	GPαv/IIIa	vitronectin, fibrinogen, fibronectin, collagen, osteopontin, thrombospondin, von Willebrand factor

## Abbreviations

AA	arachidonic acid
ACS	acute coronary syndrome
ADP	adenosine diphosphate
ATP	adenosine triphosphate
ApoER2	apolipoprotein E receptor 2
APP	amyloid precursor protein
Aβ	amyloid beta
CAD	coronary artery diseases
CCR1 (3, 4)	C-C chemokine receptor type 1 (3, 4)
CLEC	C-type lectin-like receptor
CNS	central nervous system
CVD	cardiovascular diseases
CXCL11 (12, 16)	C-X-C motif chemokine ligand 11 (12, 16)
DEP	diesel exhaust particles
DP_1_	prostaglandin D_2_ receptor 1
DVT	deep vein thrombosis
DTS	dense tubular system
EC	endothelial cells
EP_1-4_	prostaglandin E_2_ receptors 1-4
Erk	extracellular stimuli-responsive kinase
GPCR	G-protein-coupled-receptors
GPIb	glycoprotein Ib
GPO	glycine-proline-hydroxyproline
GPVI	glycoprotein VI
IL-6	interleukin-6
ITAM	immunoreceptor tyrosine-based activation motif
LAD	left anterior descending artery
LAT	linker for the activation of T cells
MI	myocardial infarction
MIF	macrophage migration inhibitory factor
MK	megakaryocytes
NMDAR	N-methyl-D-aspartate receptor
oxLDL	oxidized low density lipoprotein
PA	phosphatidic acid
PANX1	pannexin-1
PDI	protein disulfide isomerase
PGD_2_	prostaglandin D_2_
PGE_2_	prostaglandin E_2_
PGI_2_	prostaglandin I_2_
PKC	protein kinase C
PLATO	Platelet Inhibition and Patient Outcome
PLCγ2	phospholipase Cγ2
PLD	phospholipase D
Prb	probenecid
PS	phosphatidylserine
SAP	stable angina pectoris
SDF-1	stromal cell-derived factor 1, CXCL12
SFK	Src family kinases
SR	scavenger receptor
TP	TXA_2_ receptor
TxA2	Thromboxane A_2_
VLDLR	very low density lipoprotein receptor
vWF	von Willebrand factor
